# FcRL1, a New B-Cell-Activating Co-Receptor

**DOI:** 10.3390/ijms26136306

**Published:** 2025-06-30

**Authors:** Zhitao Chen, Chenxi Miao, Yan Zhang, Jiaqi Huang, Yanan Sun, Juan Chen, Jiazeng Sun, Wenbiao Shi, Xifan Wang, Ran Wang, Yixuan Li, Xingwang Zhao

**Affiliations:** 1 Key Laboratory of Precision Nutrition and Food Quality, Department of Nutrition and Health, China Agricultural University, Beijing 100083, China; 2 College of Food Science and Engineering, Gansu Agricultural University, Lanzhou 730070, China; 3 Research Center of Protection and Utilization of Plant Resources, College of Bioscience and Biotechnology, Shenyang Agricultural University, Shenyang 110866, China; 4 Department of Obstetrics and Gynecology, Columbia University, New York, NY 10032, USA

**Keywords:** FcRL1, B-cell, co-receptor

## Abstract

Fc receptor-like 1 (FcRL1), a co-receptor specifically expressed on the surface of B-cells, plays a pivotal role in modulating B-cell immune activation and orchestrating humoral immune responses. This comprehensive review systematically synthesizes research advances in FcRL1 mediated transmembrane signal transduction mechanisms, its regulatory capacity in humoral immune responses, expression patterns during B-cell differentiation and development, and expression dynamics in B-cell malignancies, while critically evaluating the therapeutic potential of FcRL1 as a cellular targeting candidate.

## 1. Introduction

Normal and well-regulated humoral immunity relies on the precise control of B-cell immune activation. B-cells, which play an indispensable role in the adaptive immune system, are the primary source of antibodies that can act to neutralize and eliminate pathogens [1]. Recent research has revealed that B-cells possess a surface decorated with more than 20 distinct immunoglobulin (Ig) superfamily receptors, each playing a pivotal role in either positively or negatively regulating the intricate process of B-cell immune activation [2].

These Ig superfamily receptors act as gatekeepers, carefully balancing the activation and suppression of B-cell responses. Some receptors, such as CD19 and CD21, enhance B-cell activation by lowering the threshold for antigen-induced signaling [3]. Conversely, other receptors, including CD22 and FcγRIIB, serve as negative regulators, preventing excessive or inappropriate B-cell activation which could otherwise lead to the development of autoimmunity or chronic inflammation [4].

The interplay between these positive and negative regulatory receptors is essential for maintaining a healthy and effective humoral immune response. By fine-tuning B-cell activation, these receptors facilitate the production of antibodies in response to foreign antigens while reducing the likelihood of generating self-reactive antibodies that could harm the body’s own tissues [5].

## 2. Characteristics of FcRL1 Molecules

**Figure 1:**
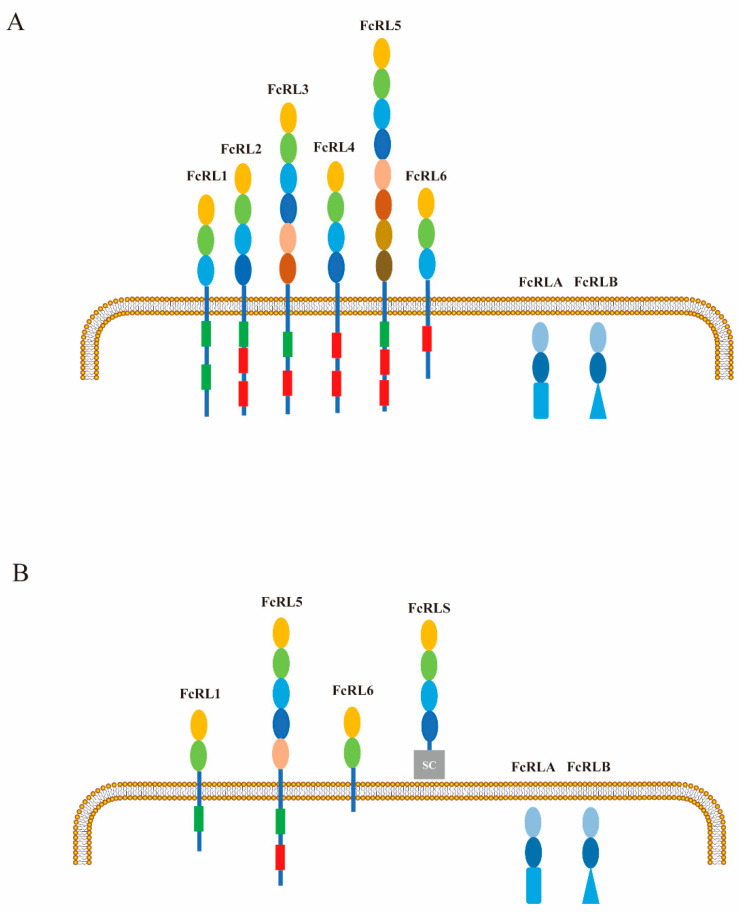
A comparative analysis of the protein properties of human and mouse FcRL molecules. The extracellular Ig domains of human (**A**) and mouse (**B**) FcRL molecules are color-coded, with red boxes representing ITIM and green boxes representing ITAM in the cytoplasm. The FcRLS type B-SRCR domain is illustrated as a gray rectangle. It should be noted that both FcRLA and FcRLB contain C-terminal mucin-like regions (blue triangles), which are expressed intracellularly.

**Figure 2:**
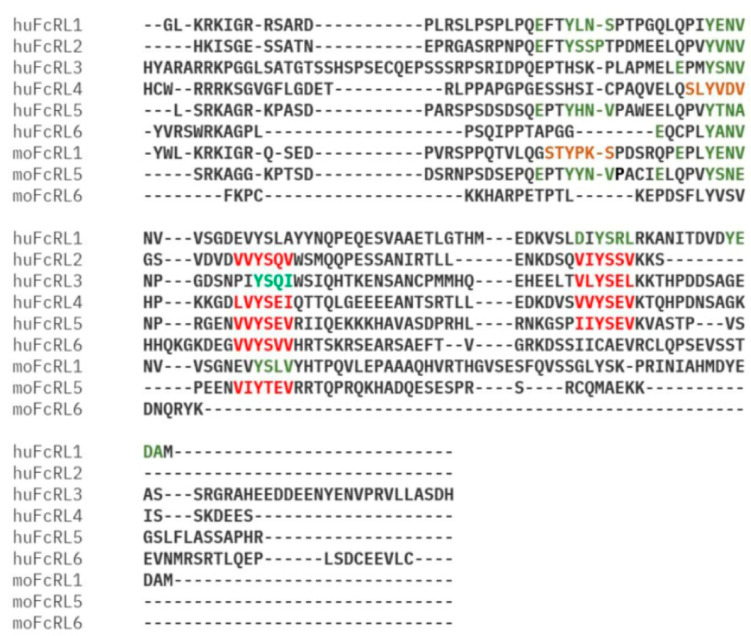
Multiple sequence alignment of human and mouse FcRL cytoplasmic tails. Multiple sequence alignment of human and murine FcRL cytoplasmic tails highlights the dual activating and inhibitory potential inherent to this receptor family. Conservative analysis was performed using CLUSTALW, with the derived consensus sequence positioned above the alignment. Key signaling motifs are annotated as follows: ITAM sequences (green), ITIMs (red), and ITSMs (yellow).

FcR-like (FcRL) molecules, a recently identified family of type I membrane proteins containing Ig domains, have been found to share a common ancestor with FcRs [6]. These proteins have garnered significant attention due to their potential role in modulating B-cell function. To date, researchers have identified eight human and six mouse members of the FcRL family. While human B-cells express FcRL1 to FcRL5 on their plasma membrane, mouse B-cells only express FcRL1 and FcRL5. Interestingly, all five human FcRL proteins (FcRL1 to FcRL5) possess at least one immunoreceptor tyrosine-based regulatory motifs activation motifs (ITAMs) or immunoreceptor tyrosine-based inhibitory motifs (ITIMs) within their cytoplasmic tails, suggesting their involvement in regulating B-cell activation (Figure 1) [6]. All members of the FCRL family (FCRL1–5) contain one or more evolutionarily conserved tyrosine-based immunoreceptor motifs in their cytoplasmic tails, suggesting conserved capacity for intracellular signal transduction upon extracellular ligand engagement. These sequence elements encompass canonical ITIMs defined as (I/V/L/S)-X-Y-X-X-(L/V/I), where X denotes any amino acid residue, and/or ITAM-related sequences with canonical variations (E/D)-X-XY-X-X-(L/I)-X6-8-Y-X-X-(L/I). A third sequence category of interest is the tyrosine-based switch motif (ITSM) characterized by the consensus T-X-Y-X-X-(V/I). While prototypical ITSM consensus sequences remain unidentified in FcRL cytoplasmic tails, putative ITSMs are observed in human FcRL4 and murine FcRL1 (Figure 2).

**Figure 3:**
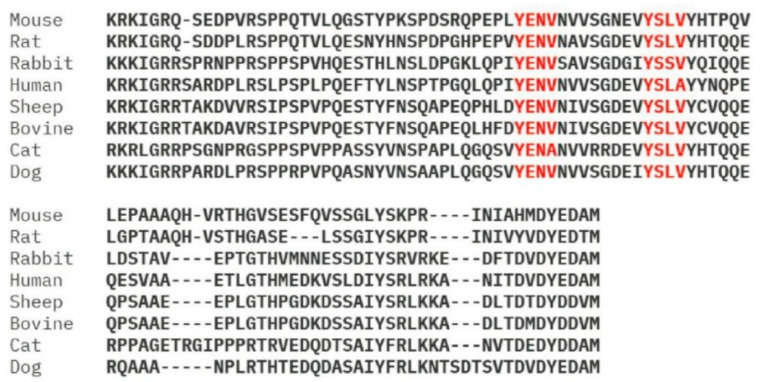
Sequence alignment of the cytoplasmic tail of FcRL1. Alignment of the amino acid sequences of FcRL1 cytoplasmic tail from different species in UniProt database. The conserved tyrosine-based motifs at positions Y_281_ENV, Y_293_SLV are noted in highlight color.

Among the FcRL family members, FcRL1 has unique structural features that hint at its potential function. It contains two ITAM-like motifs in its cytoplasmic tail (Figure 3), a glutamic acid residue with acidic properties can be found in its transmembrane region, as well as three immunoglobulin-like domains in the extracellular space [7]. Although specific ligands for FcRL1 have yet to be identified, the presence of ITAM-like motifs provides compelling evidence that it plays a role in B-cell activation. The presence of a negatively charged glutamic acid residue in the transmembrane region of FcRL1 implies that this protein may interact with other transmembrane proteins to form functional complexes, but such FcRL1-associated proteins remain to be discovered [2,7].

It is noteworthy that the amino acid sequences of two tyrosine-based ITAM-like motifs in FcRL1 exhibit a degree of variation among different species and diverge from the conventional ITAM sequence (D/E-X-XY-X-X-L/I-X6-8-Y-X-X-L/I), which is observed in B-cell receptor (BCR) subunits, namely Igα and Igβ (Figure 3) [8]. This difference in sequence may contribute to the unique signaling properties of FcRL1 and its capacity to regulate B-cell activation in response to specific stimuli.

As research on FcRL molecules advances, it has become progressively apparent that these proteins exert a pivotal influence on B-cell functionality and humoral immunity [7,9]. Understanding the complex signaling pathways mediated by FcRL1 and other FcRL family members could provide valuable insights into the formulation of targeted treatments for autoimmune disorders, B-cell malignancies, or other diseases involving aberrant B-cell activation.

## 3. Expression Pattern of FcRL1 Molecules

Recent studies have reached a consensus regarding the selective expression of FcRL1 on the membrane of CD19^+^ mature B-cells. Two novel monoclonal antibodies targeting FcRL1 have been used to demonstrate that this receptor exhibits expression in mature B-lineage cells, particularly in the context of naive and memory B-cells [7]. Interestingly, the level of FcRL1 expression varies among different B-cell subsets. The highest levels of FcRL1 were observed in naive B-cells, with intermediate levels detected in pre-germinal center (pre-GC) and memory B-cells. In contrast, the lowest levels of FcRL1 were found in germinal center (GC) B-cells and plasma cells [10,11]. These findings are consistent with the observed FcRL1 transcription levels being highest in naive B-cells, decreasing to low levels in pre-GC, GC, and plasma cells, and returning to intermediate levels in memory B-cells. In the bone marrow, the expression of FcRL1 starts at the stage of precursor B-cells and then increases as the cells differentiate, with the highest levels in mature B-cells [2,7].

The study has confirmed similar expression patterns of FcRL1. In the tonsil, the expression of FcRL1 reaches its highest level on IgD^+^CD38^−^ naive B-cells, which are mainly located in the mantle zone of the follicle. The abundant transcription of FcRL1 by cells in this region correlates with its high surface expression. As these cells become activated and differentiate into IgD^+^CD38^+^ pre-GC cells and further migrate into IgD^−^CD38^+^ GC B-cells, FcRL1 expression is down-regulated on the cell interface. However, there is an increase in the proportion of FcRL1 expressed on IgD^−^CD38^−^ memory B-cells. suggesting a potential role in maintaining B-cell memory. Interestingly, compared to memory B-cells, IgD^−^CD382^+^ plasma cells (PCs) express lower levels of FcRL1. FcRL1 expression in the spleen is also restricted to naive and memory B-cells, further supporting its role in developing and functioning B-cells [12,13].

As research on FcRL1 and other FcRL family members continues to progress, it will be essential to elucidate the specific functions of these receptors in modulating B-cell differentiation and development. Understanding the complex interplay between FcRL1 and germinal center events such as Ig class switching and somatic hypermutation may provide valuable evidence on how the abnormal activation of B-cell leads to the occurrence of B-cell functional disorder diseases, including autoimmune diseases and malignant tumors.

## 4. Molecular Mechanism of FcRL1 Regulating BCR-Mediated Immune Activation

Upon antigen exposure, BCRs undergo a complex series of signal transduction events that eventually result in B-cell activation, proliferation, and formation of an immunological synapse. The first step in this pathway is the oligomerization of BCRs into micro-clusters [14], which is associated with tyrosine phosphorylation of ITAMs located in the cytoplasmic domains of the CD79A–CD79B heterodimers [8]. This phosphorylation is mediated by Src family kinases, including Lyn, Fyn or Blk, that are rapidly active when they bind to the antigen [15].

The phosphorylated ITAMs serve as docking sites for the binding and activation of spleen tyrosine kinase (Syk), a key regulator of the BCR signaling cascade [16,17]. Then Syk phosphorylates and activates a number of downstream signaling molecules. These include phospholipase C-γ2 (PLC-γ2) [18], Bruton’s tyrosine kinase (BTK) [19], and the guanine nucleotide exchange factor (Vav). These enzymes work in concert with adaptor molecules, including the B-cell linker (BLNK) and the B-cell adaptor for PI3K (BCAP), to form a highly organized and dynamic signaling complexity known as the Syk-derived membrane-proximal signalosome [20,21].

The BCR signalosomes also recruit the p85α/p110δ isoform of phosphatidylinositol 3-kinase (PI3K), which can catalyze the transformation of phosphatidylinositol (4,5)-bisphosphate (PIP2) into phosphatidylinositol (3,4,5)-trisphosphate (PIP3) [22]. The PIP2/PIP3 balance is tightly regulated through the opposing activities of PI3K and the phosphatase and tensin homolog (PTEN). This equilibrium serves a crucial role in the modulation of B-cell activation by the guanine nucleotide exchange factor Dock2, which coordinates the remodeling of the F-actin cytoskeleton and regulates the growth and stability of BCR micro-clusters [23,24,25].

**Figure 4:**
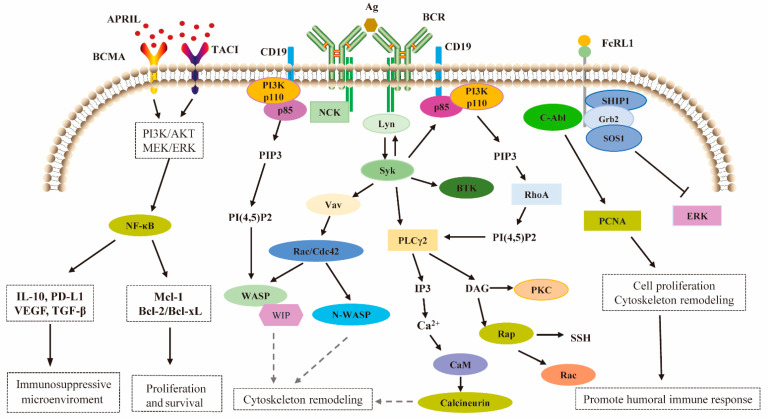
Signal transduction mediated by BCR, FcRL1, CD19, and BCMA receptors on B-cells. Upon antigen binding, BCRs oligomerize into micro-clusters, which is paralleled by the simultaneous tyrosine phosphorylation of ITAMs in the cytoplasmic domains of the CD79A–CD79B heterodimers. This phosphorylation is mediated by Src family kinases, including Lyn, which are rapidly recruited following antigen binding. The phosphorylated ITAMs provide docking sites for the recruitment and activation of Syk. Syk subsequently phosphorylates and transactivates a series of downstream signaling molecules, which include PLC-γ2, BTK, and Vav. These enzymes work together with adaptor molecules like BLNK and BCAP to constitute a highly organized and dynamic signaling complex called the membrane proximal signalosome derived from Syk. CD19 also recruits the p85α/p110δ isoform of PI3K, acting to catalyze the conversion of PIP2 to PIP3. The balance between PIP2 and PIP3 is tightly regulated through the opposing effect of PI3K and the phosphatase and tensin homolog (pPTEN). This equilibrium is critical in modulating B-cell activation and remodeling of F-actin cytoskeleton by Wiskott–Aldrich syndrome protein (WASP) and neuronal WASP (N-WASP). WASP- and N-WASP-mediated actin reassembly and reorganization are responsible for B-cell spreading and BCR cluster formation. CD19 can independently trigger intracellular Ca^2+^ flux upon ligation by associating with CD21, CD81, and Leu13 to form a signal transduction complex that functions autonomously from the BCR complex. The resulting intracellular Ca^2+^ flux regulates downstream gene expression via the calcineurin/nuclear factor of an activated T-cell (NFAT) signaling pathway. BCR cross-linking alone resulted in phosphorylation of the intracellular Y_281_ENV motif of FcRL1, providing a docking site for c-Abl, an Src homology 2 (SH2) domain-containing kinase. The c-Abl can directly interact with tyrosine 211 (Y_211_) phosphorylated proliferating cell nuclear antigen(pPCNA) to modulate cell proliferation and cytoskeletal rearrangement. pPCNA at tyrosine 211 (Y_211_) promoted its association with the non-receptor tyrosine kinase c-Abl. On the other hand, phosphorylation of the intracellular Y_281_ENV motif of FcRL1 can recruit molecular complexes such as growth-factor receptor-bound protein-2 (GRB2), son of sevenless homolog 1 (SOS1), and SH2 domain-containing inositol polyphosphate 5-phosphatase 1 (SHIP-1) and negatively regulate the phosphorylation of ERK. A-proliferation inducing ligand (APRIL) is the specific receptor for BCMA, transmembrane activator, calcium modulator, and cyclophilin ligand interactor (TACI). BCMA/TACI signaling activates NF-κB signaling and up-regulates key cell cycle regulators (e.g., CCND1/2), anti-apoptotic proteins (e.g., Mcl1, Bcl-2, Bcl-xL, BIRC3), osteoclast promoting factors (e.g., CCL3/4, SDF-1) and adhesion molecules (e.g., ICAM-1, CD44). The APRIL/BCMA/TACI signaling cascade also triggers key immunosuppressive factors (e.g., IL-10, PD-L1, TGF-β, VEGF).

The cytoskeletal rearrangements triggered by BCR signaling promote a distinctive two-phase response in B-cells [25]. Initially, the cells expand over the antigen-bearing surface, thereby maximizing the contact area and facilitating the capture of antigen by the BCRs. Then a contraction phase follows, during which the BCR and antigen-coupled micro-clusters are collected and concentrated toward the center of the B-cell immunological synapse [26]. This highly organized structure is essential for sustained signaling and efficient initialization of the antigen, which ultimately allows the antigen to be processed and presented to T-cells (Figure 4).

Our previous research using CRISPR-edited FcRL1-knockout (KO) CH-27 cells and FcRL1-KO primary B-cells of C57BL/6 mice expressing HA-tagged FcRL1 in vitro have given valuable clues to the function of FcRL1 in BCR signaling. These studies have shown that while FcRL1 receptors undergo oligomerization at the stimulation interface upon specific cross-linking, they do not directly trigger the activation phenotype of BCR molecules. Interestingly, when BCR is cross-linked alone by specific surrogate antigens, FcRL1 and BCR simultaneously oligomerize and co-localize within immune synapses [9]. These findings indicate that FcRL1 is involved in regulation of BCR immune activation in a ligand-independent manner, highlighting its potential role as a modulator of the B-cell responses.

For further investigation of the biophysical effects of mouse FcRL1 (mFcRL1) deficiency on BCR activation, total internal reflection fluorescence microscopy (TIRFM) imaging was employed with CRISPR edited FcRL1-KO CH-27 cells and FcRL1-KO primary B-cells derived from C57BL/6 mice. In these experiments, the BCR was stimulated for 10 min with F(ab’)2 anti-mouse IgM fragments that were embedded on the surface of lipid bilayers. Quantitative analysis of mean fluorescence intensities (MFI) by TIRFM revealed significantly defective synaptic accumulation of the BCR and key signaling molecules such as phosphorylated Syk (pSyk), phosphorylated BLNK (pBLNK) and phosphorylated PI3K (pPI3K, p85α subunit) not only in CH-27 cells but also in primary B-cells lacking mFcRL1. Notably, these losses could be restored by transducing with wild-type mFcRL1, highlighting the specific role of this receptor in modulating BCR signaling [9].

Apart from the impairment of synaptic accumulation of BCR and associated signaling molecules, mFcRL1 deficiency also significantly impacted calcium mobilization triggered by specific antigens. These findings are consistent with the observations of DeLuca et al. who reported decreased calcium mobilization after BCR stimulation in FcRL1-deficient 129S1/SvImJ primary B-cells and the class-switched IgG2a mouse memory B-cell line A20IIA1.6 [27]. Together, the above studies highlight the importance that FcRL1 plays in the regulation of calcium signaling downstream of the BCR.

Previous work on the interaction of human FcRL1 (hFcRL1) on the calcium signaling in FcRL1-positive Burkitt’s lymphoma (BL) cells provided additional context for the mechanistic understanding of the role of this receptor in B-cell activation [28]. While monoclonal antibody (mAb) mediated ligation of hFcRL1 alone had no effect on calcium flux, co-ligation with IgM showed an enhanced effect on the mobilization of intracellular calcium in comparison to BCR stimulation alone [9]. In summary, we found that hFcRL1 may serve as a co-stimulatory receptor that amplifies BCR-mediated calcium signaling in human B-cells.

The cumulative evidence from above studies highlights the essential function of FcRL1 for the modulation of BCR activation and downstream signaling events, like calcium mobilization in both mouse and human B-cells. The ligand-independent participation of FcRL1 in BCR signaling, as demonstrated by its co-localization with the BCR in immune synapses and its impact on the synaptic accumulation of key signaling molecules, suggests that this receptor may fine-tune B-cell responses to ensure appropriate antibody production and immune regulation. Further research into the precise molecular mechanisms by which FcRL1 influences BCR signaling and the potential implications of FcRL1 dysfunction in autoimmune disorders and B-cell malignancies may pave the way for developing targeted therapies that modulate B-cell responses in disease states.

## 5. FcRL1 Assumes the Regulatory Function Through Intracellular Tyrosine Motifs

Evidence of tyrosine phosphorylation of hFcRL1 was initially obtained by the transduction of the A20IIA1.6 B-cell line using an HA-tagged variant of the receptor.

Immunoprecipitation of HA-tagged hFcRL1 and subsequent Western blotting analyses of the cells stained with anti-FcRL1 biotinylated-Fab monoclonal antibody (mAb) fragments and cross-linked with streptavidin, indicated induction of tyrosine phosphorylation occurring at 2 minutes and 15 minutes post-stimulation [7]. Although the phosphorylation levels were lower at the later time point, these findings provided the first indication that hFcRL1 undergoes tyrosine phosphorylation upon activation.

To further identify potential phosphorylation sites within hFcRL1, we performed an alignment analysis of the amino acid sequences of the cytoplasmic tail of FcRL1 in different species using the UniProt database. This analysis identified three conserved tyrosine-based motifs located at positions Y_281_ENV, Y_293_SLV, and Y_339_EDA. These conserved motifs suggest that they may have a key regulatory role in the signaling functions performed by FcRL1 across species [9].

Our research using CRISPR-edited FcRL1-KO CH-27 cells expressing HA-tagged FcRL1 provided additional evidence for the phosphorylation of the receptor’s intracellular segment. Stimulation of either FcRL1 or the BCR independently triggered obvious phosphorylation of the FcRL1 cytoplasmic tail, highlighting the potential for FcRL1 to participate in both autonomous and BCR-mediated signaling pathways [9].

To identify the specific phosphorylated motif(s) responsible for mediating downstream signaling events, we conducted ELISA experiments that directly verified the interaction between the phosphorylated Y_281_ENV motif and the SH2 domain of the non-receptor tyrosine kinase c-Abl. This finding suggests that the Y_281_ENV motif, when phosphorylated, could be a specific docking site for the recruitment of c-Abl via its SH2 domain [9].

To confirm the importance of the Y_281_ENV motif and investigate the potential roles of the other conserved tyrosine-based motifs, we performed site-directed mutagenesis, replacing the tyrosine residues with phenylalanine in each of the three conserved motifs (Y_281_ENV, Y_293_SLV, and Y_339_EDA). Immunoprecipitation and TIRFM experiments using these mutants verified that the phosphorylation of the Y_281_ENV motif was crucial for providing a specific docking site for the c-Abl SH2 domain, thereby enabling full-length c-Abl molecules to be recruited to the FcRL1 signaling complex [9].

These findings shed light on the molecular mechanisms involved in FcRL1 signaling, revealing the relevance of tyrosine phosphorylation in the cytoplasmic tail and downstream signaling events.

The study results reported by DeLuca et al. provide further insights into the characteristics of tyrosine-based signaling associated with mFcRL1, while also highlighting discrepancies with the results obtained in our own study. Using an unbiased biochemical approach, DeLuca et al. conducted an analysis of lysates derived from pervanadate-treated FcγRIIb-deficient A20.IIA1.6 (A20) cell line that expressed FLAG-tagged wild-type mFcRL1 (WT mFcRL1) or a tail variant in which a total of six tyrosines were mutated to phenylalanine (Y_6_F). The results of the immunoprecipitation and liquid chromatography–tandem mass spectrometry (LC–MS/MS) analyses indicated the presence of phosphorylation at residues Y_281_ and Y_297_, as well as the possible binding of the protein concerned to GRB2, GRB2-related adapter protein(GRAP), SHIP-1 and SOS1 [27].

In order to investigate these interactions in more detail, DeLuca et al. transfected WT, Y_281_F, Y_297_F, and Y_6_F mFcRL1 mutants into CRISPR-edited FcRL1^−/−^ A20 cells and stimulated the samples with pervanadate. They found the Y_281_ residue was necessary for the recruitment of GRB2, SOS1, and SHIP-1. In addition, SOS-1 and SHIP-1 binding to FcRL1 are indirect and dependent on GRB2. Nevertheless, the recruitment of these effectors exhibited notable differences under conditions of BCR stimulation. Following BCR triggering, the GRB2 association remained intact, while the binding of SHIP-1 was observed to be relatively weak. Furthermore, the detection of SOS1 was no longer evident.

Given the role of GRAP and GRB2 in potentiating the phosphorylation of MAPK/ERK signaling in human B-cells, DeLuca et al. analyzed intracellular pERK activation in A20 cells with intracellular flow cytometry. After BCR stimulation, the FcRL1 deficiency significantly increased ERK activation. Dynamic trend of ERK1/2 phosphorylation in FcRL1^−/−^ A20 cells transfected with WT, Y_281_F, Y_297_F, and Y_6_F mFcRL1 mutants showed that both Y_281_ and Y_297_ were required for maximal ERK inhibition. In addition, they confirmed the results in primary splenic B-cells from WT and FcRL1^−/−^ mice and found that FcRL1 inhibited ERK in both immature and mature B-cells after BCR engagement.

It is interesting to note that no impact of FcRL1 on SyK, or PI3K phosphorylation was observed under these conditions in both A20 cells and primary splenic B-cells, which is inconsistent with our previous findings of impaired synaptic accumulation of pSyk, pPI3K , and pBLNK in FcRL1-deficient cells [9,27].

The above results suggest that FcRL1 appears to mediate two distinct intracellular signaling pathways, potentially influenced by differences in the isotype of the BCR. The membrane immunoglobulin M (mIgM) is expressed on primary B-cells and CH-27 cells; conversely, A20IIA1.6 B-cells exhibit mIgG class switching. Due to their unique intracellular amino acid motifs, mIgG immunoglobulins exhibit a completely different signal transduction kinetic mechanism compared to IgM immunoglobulins [29,30]. Therefore, the elements recruited to the mFcRL1 tail may vary depending on stimulus type and strength.

In our stimulation system, the surrogate antigen, which is in a two-dimensional mobile state, is incorporated on the surface of a lipid bilayer [9]. In contrast, DeLuca et al. conjugated the surrogate antigen with beads, providing an immobile antigen stimulation [27]. The stimulation intensity and dynamics of these two approaches on B-cells differ, potentially explaining the discrepancy in signal transduction molecules recruited by the intracellular segment of FcRL1 [31,32,33].

Despite these differences, the generalized defects in humoral antigen-specific responses that were observed in two independently developed FcRL1-/- models, together with the effects on both proliferation and calcium signaling, strongly suggest a positive role for mFcRL1 in B-cells [9,27]. This notion is further confirmed by the findings of Zahra et al., who revealed that FcRL1-KO significantly reduced cell proliferation, enhanced the apoptosis, and triggered the G1 cell cycle arrest in B-cells derived from diffuse large B-cell lymphoma (DLBCL) patients. In addition, the levels of p65 NF-κB and PI3K/pAkt were clearly reduced following knockdown of FcRL1 expression [34].

Taken together, these observations suggest that FcRL1 represents an essential modulator of B-cell behavior whose intracellular signaling pathways are influenced by factors such as BCR isotype and the nature of the antigenic stimulus. Further research should be conducted to elucidate the precise mechanisms by which FcRL1 integrates and modulates these signaling pathways in the context of different BCR isotypes and stimulation conditions.

## 6. FcRL1 Regulates the Humoral Immune Response of B-Cells

In our previous research, we investigated the potential involvement of FcRL1 in GC-mediated antibody responses by vaccinating mice with sheep red blood cells and evaluating GC formation in FcRL1-KO and WT control mice. Flow cytometry showed fewer GC B-cells in FcRL1 KO mice than in WT mice. To examine the T-cell-dependent antibody response further, both groups of mice were immunized against the T-cell-dependent model antigen NP32-KLH in the presence of an aluminum-containing adjuvant. FcRL1-KO mice had significantly lower levels of NP-specific IgM (detected by NP8-BSA), high-affinity NP-specific IgG (detected by NP8-BSA) and total NP-specific IgG (detected by NP30-BSA) compared to WT mice. Enzyme-linked immunosorbent assays were used to count NP-specific IgM or IgG-producing cells from the two groups of mice to assess the influence of FcRL1 deletion on the extra-follicular plasmablasts reaction. A consistent finding was that the absence of FcRL1 resulted in a reduction in the quantity of antigen-specific extra-follicular plasmablasts. To investigate the functionality of FcRL1 in T-cell-independent antibody reactions, two groups of mice were immunized against the model T-cell-independent antigen NP50-Ficoll in combination by means of an aluminum-containing adjuvant: one group was FcRL1-KO and the other WT. ELISA experiments were performed for quantification of NP-specific IgM and the level of IgG3 (measured by NP30-BSA), which revealed that FcRL1 insufficiency significantly influenced T-cell-independent antibody responses. In conclusion, these findings substantiate the assertion that FcRL1 is instrumental in facilitating T-cell-dependent and T-cell-independent antibody responses [9].

## 7. The Aberrant Expression of FcRL1 Observed in Patients with B-Cell Malignancy

The relationship between the abnormal expression of the FcRL1 gene and B-cell-related diseases is the subject of an increasing number of research studies conducted in the last few years. These studies have begun to elucidate the potential contribution of FcRL1 to B-cell malignancy. Xing et al. confirmed the expression of FcRL1 on chronic lymphocytic leukemia (CLL), follicular lymphoma (FL), hairy cell leukemia (HCL), and mantle cell lymphoma (MCL) by flow cytometry [34]. Zahra et al. revealed higher levels of FcRL1 expression in DLBCL, HCL, and BL patients compared with control groups. There was a significant reduction in the levels of FcRL1 expression in chronic CLL and MCL patients compared with healthy individuals [35]. Tohid et al. reconfirmed this result, and they found that the corresponding mRNA expression levels of FcRL1 were significantly lower in B-CLL cases compared to normal-aged subjects. In addition, their further research indicated significant down-regulation of FcRL1 genes in ALL compared to normal subjects [36].

These findings collectively demonstrate that although FcRL1 exhibits heterogeneous expression profiles across B-cell neoplastic subtypes, its persistent expression signature supports its viability as a therapeutic target for B-cell malignancies. Xing Du constructed anti-FcRL1 immunotoxin and evaluated the specific cytotoxicity for the treatment of FcRL1-positive malignancies, including CLL, HCL, FL, MCL, and other B-non-Hodgkin lymphomas (NHLs) in vitro.

While investigations into the correlation between FcRL1 expression levels and pathological progression of B-cell malignancies remain understudied, emerging evidence delineates its mechanistic underpinnings in malignancies. A preliminary synthesis of current findings reveals FcRL1’s functional imperatives in sustaining proliferative signaling and conferring anti-apoptotic resilience during B-cell neoplastic maintenance. Zahra et al. demonstrated through BL cell-based investigations that the knockdown of FcRL1 was observed to markedly reduce cell proliferation and increase apoptotic cell death. The anti-apoptotic gene Bcl-2 was significantly down-regulated in FcRL1-knockdown BL cells, whereas the pro-apoptotic genes BID and BAX were up-regulated [35]. Furthermore, the activation of PI3K/p-AKT and phosphorylated p65 NF-κB, which are key signaling pathways associated with cell survival and reproduction, was markedly down-regulated in FcRL1-knockdown BL cells in contrast to control cells. Our experimental data revealed that genetic ablation of FcRL1 significantly impairs the antigen recognition-triggered proliferation of primary B-cells. Furthermore, we identified that the phosphorylated Y_281_ENV motif within FcRL1’s intracellular domain serves as a molecular scaffold for c-Abl recruitment. As a nonreceptor tyrosine kinase, c-Abl is involved in both the regulation of cytoskeletal remodeling and cell proliferation and V(D)J recombination during B-cell development. In addition, numerous studies have proved that c-Abl is a modulator of P53.

Nearly three decades ago, Nancy L. Nicolson’s research demonstrated that nuclear protein complex fractions (NPCF) isolated from normal leukocyte nuclei contained minimal detectable levels of tightly bound c-Abl, p53, or Bcl-2 genes or genomic sequences. In contrast, nuclei from chronic myeloid leukemia (CML) cells frequently exhibited tight associations of these genes with multiple NPCFs. Examination of NPCF isolated from the leukocyte nuclei from patients with highly progressive CML for the presence of the three genes revealed that more NPCF contained the three tightly bound genes than leukocyte NPCF from patients with stable or less progressed CML [37].

Taken together, these findings collectively elucidate that FcRL1 orchestrates critical oncogenic programs by not only potentiating tumor cell proliferation but also reinforcing apoptosis resistance.

## 8. Similarities and Differences Between FcRL1 and Other FcRLs in B-Cell Functional Modulation

Although FcRL1, FcRL2, FcRL3, FcRL4, and FcRL5 are all expressed on the surface of memory B-cells [13,38,39,40,41], the latter four members exhibit significant differences from FcRL1 in their regulatory effects and underlying mechanisms on B-cell function. The ligands for human FcRL1 and FcRL2 remain unidentified. FcRL3 serves as a receptor for secretory IgA (SIgA), FcRL4 specifically binds dimeric IgA, and FcRL5 interacts with intact IgG [42] (Table 1).

**FcRL2**

FcRL2 contains two canonical ITIMs and a putative ITAM sequence within its cytoplasmic domain [49]. Mutagenesis analysis reveals that tyrosine residues within both inhibitory motifs are strictly required for complete suppression of BCR signaling, rather than the tyrosine residue within the putative activation motif. The phosphorylated tyrosine residue (Y_502_) within the ITIM motif directly recruits and interacts with SH2 domain-containing phosphatase 1 (SHP-1). Although anti-FcRL2 stimulation alone failed to alter ERK phosphorylation, the co-ligation of the BCR and FcRL2 potently suppressed ERK1/2 phosphorylation, establishing FcRL2 as a negative immune-regulatory function in memory B-cells [38].

While FcRL2 expression is up-regulated in CLL, MCL, BL, and multiple myeloma (MM) [11,50,51]. Nückel et al. found a paradoxical inverse relationship between FcRL2 mRNA expression and disease progression in CLL. The researchers analyzed FcRL2 mRNA expression in a large cohort of 152 CLL patients to evaluate its potential as risk prediction for B-cell CLL. Quantitative analysis revealed significantly elevated FcRL2 mRNA levels in B-CLL patient-derived peripheral blood mononuclear cells (PBMCs) compared to healthy controls (1.35–210-fold up-regulation, *p* < 0.0001). Patients with high FcRL2 expression (as defined by ROC analysis) showed significantly better outcomes when compared to those with low expression. The median treatment-free survival (TFS) was 119 months for the high-expression group versus 34 months for the low-expression group (*p* < 0.0001). Regarding overall survival (OS), the median was 321 months for the high-expression group, while it was not reached for the low-expression group (*p* = 0.009) [52].

Compared with other indicators, FcRL2 was also superior at predicting the time to first therapy in CLL. Patients with high FcRL2 expression had a median treatment-free interval of 15.5 years versus 3.75 years for low expressers [50].

These clinical findings further demonstrate that FcRL2-mediated suppression of BCR signaling exerts protective effects against disease progression in B-cell malignancies.

**FcRL3**

FcRL3, upon tyrosine phosphorylation, serves as a docking platform for both SH2 domain-containing tyrosine kinases and tyrosine phosphatases. Site-directed mutagenesis studies demonstrated that ITAM-embedded tyrosine residues 650 and 652 (Y_650_/Y_662_) specifically mediate Syk and ZAP-70 binding, and ITIM-localized tyrosine residues 692 and 722 (Y_692_/Y_722_) are critical for SHP-1 and SHP-2 interactions. This demonstrates FcRL3’s dual functionality in signal regulation [39,53].

Research demonstrates that FcRL3 overexpression or co-ligation with BCR potently suppresses BCR signaling in a GC-derived cell line. Cui et al. reveals that FcRL3 activates both the SHP-1 phosphatase and p38 MAPK signaling pathways in B-cells, thereby promoting IL-10 secretion [54]. This FcRL3-driven IL-10 production enhances regulatory T-cell (Treg) functionality, ultimately leading to broad suppression of pro-inflammatory cytokine secretion.

Li et al. discovered that FcRL3 ligation enhances CpG oligodeoxynucleotide TLR9-mediated B-cell responses, including proliferation, activation, and survival, yet exerts contrasting suppression of plasma cell differentiation and antibody production [39]. Whether the molecular mechanism underlying these observations involves FcRL3’s intracellular ITAM motifs requires further investigation and validation. While FcRL3 is overexpressed in CLL and FL [11,12,50,51], its pathological mechanisms in B-cell malignant transformation and progression remain poorly characterized.

**FcRL4**

FcRL4’s cytoplasmic domain contains three critical tyrosine residues: Y_451_ located in a membrane-proximal switch motif and Y_463_/Y_493_ embedded within ITIMs [55]. Under normal physiological conditions, FcRL4 achieves the recruitment of SHIP and SHP-1/SHP-2 through the phosphorylation regulation of three tyrosine sites in the intracellular segment, thereby suppressing BCR-mediated downstream signal transduction, including down-regulation of Syk phosphorylation, blockade of PLC-γ2 and Vav activation, suppression of calcium mobilization and inhibition of CD69 expression28. Moreover, FcRL4^+^ B-cells exhibit significantly elevated CD20 expression relative to other B-cell subsets [56]. Collectively, these findings demonstrate the inhibitory regulatory role of FcRL4.

FcRL4 has positive expression in different proportions in mucosa-associated lymphoid tissue (MALT) lymphoma, DLBCL, DLBCL not otherwise specified (DLBCL-NOS), central nervous system (CNS) DLBCL, primary mediastinal large B-cell lymphoma (PMBL), and B-ALL/acute lymphoblastic lymphoma (LBL) [57], but its expression is up-regulated in MZL, CLL, and FL [51,58]. Based on current evidence, it remains undetermined whether FcRL4 overexpression drives the progression of B-cell malignancies. However, studies at the molecular mechanism level revealed that when in the intracellular segment is phosphorylated simultaneously FcRL4 retained its capacity for SHP-2 binding but lost affinity for the SHP-1 and SHIP phosphatases while gaining the ability to bind PLC-γ2 [55]. This suggests that the intracellular segment of FcRL4 has the potential to mediate dual regulatory effect. Currently, there is a lack of exact data on the phosphorylation status of the FcRL4 intracellular segment in B-cell malignancies. Therefore, whether FcRL4-mediated recruitment of PLC-γ2 can promote proliferation and anti-apoptotic effects in malignant B-cells requires further investigation.

**FcRL5**

FcRL5 serves as a distinctive marker for splenic marginal zone (MZ) B-cells and peritoneal B-1 B-cells, featuring both an ITAM-like motif and a canonical ITIM sequence [59]. Lyn kinase binds to the ITAM-like tyrosine residue (Y_543_) of FcRL5, while SHP-1 is recruited to the ITIM motif at Y_566_ [60]. The functional divergence of FcRL5 between MZ B-cells and B-1 B-cell subsets directly correlates with their distinct intracellular concentrations of SHP-1 [61]. In splenic MZ B-cells, BCR signal transduction is active, but the intracellular SHP-1 level is significantly higher. When BCR undergoes co-crosslinking with FcRL5, the abundant SHP-1 is recruited to the ITIM of FcRL5, thereby inhibiting BCR stimulation—as evidenced by diminished calcium mobilization and reduced ERK phosphorylation. In peritoneal B-1 B-cells, BCR signal transduction is relatively weak (“attenuated activation”). Moreover, the abundance of SHP-1 is relatively low, approximately half that of MZ B-cells. While FcRL5’s ITIM motif can recruit SHP-1, its non-canonical ITAM-like simultaneously recruits Lyn kinase, creating a biphasic regulatory effect. This results in insufficiently potent BCR inhibition, coupled with Lyn-mediated fine-tuning of activation signals [60].

FcRL5 demonstrates significant overexpression across multiple B-cell malignancies, including MM, CLL, MCL, BL, and HCL [11,12,62,63]. Quantitative analysis revealed high soluble FcRL5 levels were specifically detected in 21/43 (49%) of patients with MM, 36/46 (78%) with CLL and 9/24 (38%) with MCL [63]. These clinical observations strongly suggest that FcRL5 up-regulation may functionally contribute to the pathogenesis of B-cell malignancies.

Recent studies demonstrate that in the presence of T-cell help and TLR9 agonists stimulation, co-ligation of FcRL5 and BCR on naive B-cells modulates calcium signaling, enhances B-cell proliferation and drives immunoglobulin class-switching from IgM to IgG/IgA [64].

While human peripheral B-cells express either FcRL5 or CD21, Franco et al. identified a substantial subset of tonsillar B-cells that co-express both receptors [65]. Using conventional biochemical techniques, they discovered that FcRL5 constitutively interacts with CD21. Notably, the FcRL5–CD21 complex assembled efficiently in CD19-deficient conditions, confirming CD19 as dispensable for this molecular association. Moreover, FcRL5 cross-linking induced rapid CD19 recruitment to the FcRL5. Nevertheless, CD19–FcRL5 complexes were observed to form in the absence of CD21. Further results revealed that engagement of FcRL5 triggers rapid recruitment of key B-cell signaling molecules, including CD19, activated PLC-γ2, and BTK, implicating a novel activating function for FcRL5 [65].

Triple co-engagement of FcRL5, CD21, and BCR induced a markedly enhanced calcium influx compared to dual ligation of CD21 and BCR alone [65].

Collectively, these results demonstrate that FcRL5 exhibits dual signaling capacity, wherein CD21 co-engagement acts as a molecular switch to convert FcRL5 from an inhibitory to an activating co-receptor. This functional plasticity endows FcRL5 with the potential to drive tumor progression.

All findings collectively demonstrate that the diversity of tyrosine-based motifs within the intracellular domains of FcRLs confers diversified effector mechanisms. We can no longer simply assume that in terms of the regulation of B-cell function, except for FcRL1 exerting a positive regulatory effect, FcRL2–5 all exert a negative regulatory effect. More studies from diverse perspectives and in different physiological conditions need to be further carried out to elaborate the functions and effect mechanisms of FcRLs more comprehensively. From the perspective of molecular mechanisms, compared with other FcRLs that recruit SHP-1/2 through direct interactions, FcRL1 recruits the negative regulatory molecule SHIP-1 through indirect interactions. The recruitment ability of the intracellular segment of FcRL1 for phosphokinase molecules that exert the positive regulatory effect of BCR signal transduction, such as Syk and Lyn, still requires further investigation. We have systematically summarized the expression patterns of FcRLs in different B-cell subtypes and different diseases, as well as the regulatory factors of expression, as shown in Table 2.

## 9. The Potential of FcRL1 to Become a New Chimeric Antigen Receptor T-Cell (CAR-T) Target for the Treatment of B-Cell Malignancies

The potential of FcRL1 as a novel CAR-T target hinges on two pivotal biological determinants: its lineage-restricted expression specificity and biological features manifested during B-cell activation trajectories. CAR-T therapeutics targeting FcRL1 have remained unreported in both preclinical and clinical domains, where CD19 and BCMA continue to dominate as gold-standard targets with validated clinical efficacy [90,91]. By comparing and analyzing the similarities and differences in the functional mechanisms of FcRL1 with CD19 and BCMA, a forward-looking inference can be made regarding the feasibility of FcRL1 as a cell therapy target.

During the initiation phase of B-cell immune activation, as BCRs aggregate at the antigen-presenting interface to orchestrate immunological synapse (IS) assembly, CD19 and FcRL1 are concomitantly integrated into the synapse architecture [92,93]. Notably, no direct experimental evidence has yet elucidated BCMA’s participation in this molecular choreography of immune synapse formation. The current research progress indicates that BCMA plays a significant role in maintaining the survival and differentiation of B-cells and exerts a crucial driving effect on the proliferation of B-cell tumor cells. The mechanistic architecture underpinning FcRL1, CD19, and BCMA effector functions on B-cells is schematized in (Figure 4).

Compared with CD19, current research evidence endows FcRL1 with two potential advantages. Firstly, both Jeffrey A. Ledbetter and Ian Krop’s research evidence suggesting that CD19 can independently induce intracellular Ca2+ flux upon ligation by associating with CD21, CD81, and Leu13 to generate a signaling composite that functions separately from BCR complex [94,95]. The resulting intracellular Ca^2+^ flux regulates downstream gene expression via the calcineurin/NFAT signaling pathway. CD19-mediated NFAT signaling has been reported to have a critical function in the regulation of genes such as Igκ, TNF-α, CD5, c-Jun, IL-6, and IL-10, providing feedback to regulate the biological phenotype of B-cells and contributing to the survivability of activated B-cell DLBCL (ABC DLBCL) [96,97,98]. In contrary, Chuen-Miin Leu’s research findings indicate that cross-linking of FcRL1 is insufficient to induce Ca^2+^ flux [7,9]. Consequently, CAR-T cells may evoke discriminatory responses in B-cells during their recognition of CD19 versus FcRL1, given that research indicates the initiating phase of CAR-T therapy involves recognizing and ligating the CAR receptor with its corresponding ligand on the surface of the target cell. Upon intracellular signaling, the receptors converge on the surface of the effector cell and assemble at the interface of both cells, forming the IS [93,94]. This mechanistic insight posits that FcRL1 as a CAR recognition target may reduce abnormal gene expression caused by the triggering of intracellular calcium flow, such as the loss of target antigens and other issues.

Secondly, findings from single-cell transcriptomic profiling reveal that CD19’s expression within Brain mural cells, indicating that it is not expressed strictly and specifically on the surface of B-cells. In sharp contrast, FcRL1 demonstrates a high degree of B-cell lineage specificity. To date, there is no evidence of FcRL1 expression on cells other than B-cells. This distinct expression pattern strongly implies that FcRL1-targeted CAR-T cells might avoid CD19-related immune effector cell-mediated neurotoxicity syndromes (ICANS) via increased target selectivity [99].

In comparison to BCMA, FcRL1’s most prominent advantage lies in the fact that, to date, there is no evidence suggesting the existence of a soluble form of FcRL1. In sharp contrast, γ-secretase-mediated ectodomain shedding gives rise to soluble BCMA (sBCMA), which competitively interferes with the engagement of CAR molecules by sequestering ligand–receptor interactions. This binding impairs the capacity of CAR-T cells to recognize and attach to BCMA molecules present on the surface of tumor cell membranes. Consequently, the tumor-killing effectiveness of CAR-T cells is decreased, and simultaneously, tumor resistance is augmented. Currently, CAR-T therapies with CD19 and BCMA as targets have shown notable tumor resistance in clinical applications. The causes are outlined in Table 3.

In summary, being a co-receptor specifically located on the surface of B-cells, FcRL1 can not only function as a new CAR-T target but also holds potential biological advantages regarding the avoidance of side effects.

## 10. Conclusions and Future Directions

FcRL1 is firmly recognized as a novel co-receptor that demonstrates expression specificity restricted to B-cells. Based on the current research advancements in murine models, FcRL1 has a positive regulatory function in regulating the proliferative homeostasis of B-cells and coordinating both T-cell-dependent and T-cell-independent humoral immune responses [9]. The current cellular-level investigations have identified the phosphorylated intracellular Y_281_ENV motif within FcRL1 as a critical signaling module capable of recruiting c-Abl and GRB2 to provide mechanistic insights into FcRL1-mediated transmembrane signal transduction. There remain two critical, unresolved questions that require further research for elucidation.

On one hand, additional tyrosine motifs are present within its intracellular domain. During BCR-mediated immune activation, these tyrosines, phosphorylated to varying degrees, recruit diverse intracellular signal transduction molecules, thus contributing to the fulfillment of the FcRL1 receptor’s function. Systematically delineating these latent interactomes and their spatiotemporal interaction dynamics is still an unaddressed mechanism that requires in-depth biochemical exploration.

On the other hand, it remains a question meriting in-depth exploration whether the adaptor molecules recruited by the intracellular domain of FcRL1 are consistent during the activation of different B-cell subtypes, such as IgM-BCR, IgG-BCR, IgA-BCR, and IgE-BCR. The expression patterns of FcRL1 in various B-cell malignancy subtypes have been ascertained. Specifically, its expression is elevated in DLBC, HCL, and BL, while it is decreased in CLL, MCL, and ALL. The unaddressed mechanistic foundation for FcRL1’s selective expression regulation among different neoplastic subtypes, along with the pathological implications of its expression dysregulation during tumor progression, highlight the necessity to systematically analyze the downstream transcriptional programs mediated by the FcRL1 receptor pathway. To date, FcRL1-targeted antibody-based therapies and cellular immunotherapies remain conspicuously absent from both preclinical animal models and clinical trial landscapes.

While the therapeutic merits and liabilities of FcRL1 as a molecular target demand extensive future validation, its lineage-restricted expression specificity in B-cells and widespread expression in different B-cell tumor subtypes endows FcRL1 as a novel candidate therapeutic target for next-generation antibody therapy and CAR engineering.

## Figures and Tables

**Table 1:** Molecular characteristics and expression patterns of human FcRLs in B-cells and associated immune tissues.

Receptor	Designated CD	Identified Ligands	Soluble Isoforms	Protein Expression	mRNA Expression	Refs.
**FcRL1**	CD307a	Not identified	Transmembrane	BM: pro-B-cells; pre-B-cells; nBC; MBC	Tonsil: nBC; pre-GC; MBC; GC; PC	[7,11,13,43]
				Blood: nBC; MBC		
				Tonsil: nBC (follicular mantle); GC; MBC; pre-GC; PC (low level)		
				Spleen: CD38^−^ B-cells; nBC; MBC; MZ; FO B-cells		
				LN		
**FcRL2**	CD307b	Not identified	Transmembrane	Blood: CD20^+^CD27^+^ MBC	1. Tonsil: GC light zone, intraepithelial and interfollicular regions 2. Mantle zones and slightly outside the mantle zone from tonsil: nBC-rich and MBC-poor	[2,11,12]
				Tonsil: CD138^+^CD38^++^ Pc (low level); CD20^+^IgD^−^CD38^−^ MBC		
				Spleen: CD20^+^IgD^−^CD38^−^ MBC		
**FcRL3**	CD307c	Secretory IgA	Transmembrane	BM: MBC (low level)	Tonsil: Light zone of the GC; Follicular mantle zones	[2,11,44]
				Blood: B-cells; CD27^+^ MBC; Circulating innate-like MZ		
				Tonsil: nBC (low level); MBC; GC		
				Spleen: nBC; MBC; CD21^high^CD23^low^ MZ		
				Peritoneal: B220^+^CD5^+^ B1a and B220^+^CD5^−^ B1b cells		
**FcRL4**	CD307d	Heat-aggregated IgA	Transmembrane	BM; Blood: Very low frequency	Tonsil: MBC; nBC	[45,46]
				Tonsil: IgD^−^/CD38^−^ MBC; B-cells (underneath and within the tonsil epithelium)		
				Spleen: Low frequency in MZ		
				Dome epithelium of Peyer patches; Monocytoid B-cells in reactive LNs; MLN (very low frequency)		
**FcRL5**	CD307e	All heat-aggregated IgG subtypes	Secretory; GPI-anchored; Transmembrane	BM: PC	Tonsil: Interfollicular and intraepithelial regions (rich in MBC); Centrocyte-rich light zones of GC; Follicular mantle zones (low level)	[2,11,12]
				Blood: nBC; MBC		
				Tonsil: nBC; MBC; PC		
				Spleen: nBC; MBC; CD38^++^/CD138^+^ PC		
**FcRLA**	-	IgM; IgG; IgA	Soluble	BM: pre-B-cells	1. Tonsil: pre-GC; GC 2. Spleen 3. LN	[6,47,48]
				Blood: B-cells		
				Tonsil: B-cells; PC (low level); Large CD20^+^ GC centroblast-like B-cells		
				Spleen: GC; MZ		
**FcRLB**	-	Not identified	Soluble	BM; Spleen; Tonsil: GC	Spleen; Tonsil	[6,47,48]

nBC: naïve B-cell; MBC: memory B-cell; PC: plasma cell; BM: bone marrow; MLN: mesenteric lymph node; LN: lymph node; FO B-cells: follicular B-cells; MZ: marginal zone B-cells; GC: germinal center B-cells.

**Table 2:** Abnormal expression patterns and driving factors of FcRLs in diseases.

Receptor	Diseases	Overexpression	Down-Regulation	Expression Regulated by	Refs.
**FcRL1**	Autoimmune	MS; Takayasu’s arteritis; Lupus anticoagulants; Von Willebrand	SLE; HT; GD	HBV infection	[66,67,68,69,70]
	Malignancies	NHL (FL; HCL; BL; DLBCL); Pediatric retinoblastoma; Pediatric neuroblastoma; Pediatric kidney tumor; Pediatric diffuse astrocytic and oligodendro tumor	ALL; CLL; MCL		[11,12,34,35,36,66]
	Infectious	AHB; HBV	HCV-MC vasculitis (CD21^−/low^IgM^+^CD27^+^ MZ)		[68,70,71]
**FcRL2**	Autoimmune	HT; GD	IgAN	IGHV mutation status	[68,69,72]
	Malignancies	NHL (CLL; MCL; BL); MM	-		[11,50,51]
	Infectious	HCV-MC vasculitis (CD21^−/low^IgM^+^CD27^+^ MZ); Malaria; HIV	-		[11,71]
**FcRL3**	Autoimmune	RA; SLE; AITD; pSS; GD	IgAN; MS	1. TLR9 stimulation 2. 169 C/T single nucleotide polymorphism (SNP) 3. CHB infection	[69,72,73,74]
	Malignancies	CLL; FL; SKCM; BRCA	-		[11,12,50,51]
	Infectious	Malaria individuals (AtMs); Malaria (hyposensitive atypical MBCs); HCV-MC vasculitis (CD21^−/low^IgM^+^CD27^+^ MZ); CHB	-		[71,75,76,77]
**FcRL4**	Autoimmune	GD; pSS	RA	1. BCR and TLR9 co-stimulation 2. HIV gp120 protein 3. TGF-β1 4. CHB infection	[51,66,68,69,73]
	Malignancies	MZL; CLL; FL	-		[46,51,58]
	Infectious	Malaria individuals (AtMs); HCV (CD27^−^CD21^−^ B-cells); HIV; CHB	-		[76,77,78,79,80,81]
**FcRL5**	Autoimmune	SLE; RA	-	1. Sustained BCR stimulation 2. EBV infection 3. HBV infection	[42,77]
	Malignancies	MM; CLL; MCL; BL; HCL	-		[11,12,62,63]
	Infectious	Malaria individuals (atypical MBCs); HCV-MC vasculitis (CD21^−/low^IgM^+^CD27^+^ MZ); HBV	-		[71,75,77,79]
**FcRL6**	Autoimmune	-	RA; SLE; ITP	1. PD-1 directed immunotherapy 2. HIV-1 infection	[82]
	Malignancies	CLL; LUAD; SKCM; BRCA	AML; CML		[51,82,83,84,85]
	Infectious	HIV-1^+^ patients; Malaria	-		[85,86]
**FcRLA**	Autoimmune	RA	-	CHB infection	[87]
	Malignancies	NHL (FL; MCL; MZL; CLL; BL); RCC	MM		[51,88]
	Infectious	CHB	-		[77]
**FcRLB**	Malignancies	Colorectal cancer	-	CHB infection	[89]
	Infectious	Malaria; CHB	-		[76,77]

MBC: memory B-cell; MZ: marginal zone B-cell; HT: Hashimoto thyroiditis; GD: Grave’s disease; SLE: systemic lupus erythematosus; RA: rheumatoid arthritis; MS: multiple sclerosis; pSS: primary Sjogren’s syndrome; HCL: hairy cell leukemia; CLL: chronic lymphocytic leukemia; FL: follicular lymphoma; MCL: mantle cell leukemia; MZL: marginal zone lymphoma; AtMs: atypical memory B-cells; HCV: hepatitis C virus; HCV-MC: HCV-associated mixed cryoglobulinemia; AHB: acute hepatitis B; IgAN: IgA nephropathy; AITD: autoimmune thyroid disease; NHL: non-Hodgkin’s lymphoma; MM: multiple myeloma; BL: Burkitt lymphomas; HBV: hepatitis B virus; LUAD: lung adenocarcinoma; SKCM: cutaneous melanoma; BRCA: breast carcinoma; RCC: renal cell carcinoma; AML: acute myeloid leukemia; CML: chronic myeloid leukemia; ITP: idiopathic thrombocytopenia purpura; CHB: chronic hepatitis B virus.

**Table 3:** The most frequently mentioned causes of tumor resistance to CD19 or BCMA CAR-T therapy.

Target	Cause		Tumor Type	Refs.
**CD19**	Loss of CD19	Missense and frameshift mutations in exon 2 of CD19	B-ALL	[100]
		Alternative splice variants lacking exon 2 or exons 5–6 of CD19	B-ALL	[101]
		Presence of intron 2 in mature CD19 mRNA	Lymphoma; B-ALL	[102]
		Hypermethylation of the CD19 promoter and decreased CD19 expression	CLL;BL	[103]
	Mask of CD19	CD19-targeted therapy with tafasitimab	r/r DLBCL	[104]
	Lineage switch	CD19 loss and CD34, CD33, or CD64 markers expression	Mixed lineage leukemia (MLL)-rearranged B-ALL; Philadelphia chromosome-positive B-ALL	[105,106,107,108]
**BCMA**	Induction of immunosuppressive microenvironment	APRIL/BCMA signaling cascade induces the expression of anti-apoptotic genes (Bcl-2/Bcl-xL, Mcl-1) and immune regulatory genes (IL-10, PD-L1, VEGF, TGF-β)	MM	[109,110]
	sBCMA	sBCMA compete to combine anti-BCMA CAR-T	MM	[111,112,113,114]

## Data Availability

All data needed to evaluate the conclusions in the paper are present in the paper. Additional data related to this paper may be requested from the authors.

## References

[1] Walzik D., Belen S., Wilisch K., Kupjetz M., Kirschke S., Esser T., Joisten N., Schenk A., Proschinger S., Zimmer P. (2024). Impact of Exercise on Markers of B Cell-Related Immunity: A Systematic Review. J. Sport Health Sci..

[2] Miller I., Hatzivassiliou G., Cattoretti G., Mendelsohn C., Dalla-Favera R. (2002). IRTAs: A New Family of Immunoglobulinlike Receptors Differentially Expressed in B Cells. Blood.

[3] Fearon D.T., Carroll M.C. (2000). Regulation of B Lymphocyte Responses to Foreign and Self-Antigens by the CD19/CD21 Complex. Annu. Rev. Immunol..

[4] Tsubata T. (2019). Inhibitory B Cell Co-Receptors and Autoimmune Diseases. Immunol. Med..

[5] Noor A.A.M., Nor A.K.C.M., Redzwan N.M. (2024). The Immunological Understanding on Germinal Center B Cells in Psoriasis. J. Cell. Physiol..

[6] Davis R.S. (2007). Fc Receptor-like Molecules. Annu. Rev. Immunol..

[7] Leu C.-M., Davis R.S., Gartland L.A., Fine W.D., Cooper M.D. (2005). FcRH1: An Activation Coreceptor on Human B Cells. Blood.

[8] Reth M. (1989). Antigen Receptor Tail Clue. Nature.

[9] Zhao X., Xie H., Zhao M., Ahsan A., Li X., Wang F., Yi J., Yang Z., Wu C., Raman I. (2019). Fc Receptor-like 1 Intrinsically Recruits c-Abl to Enhance B Cell Activation and Function. Sci. Adv..

[10] Pascual V., Liu Y.J., Magalski A., de Bouteiller O., Banchereau J., Capra J.D. (1994). Analysis of Somatic Mutation in Five B Cell Subsets of Human Tonsil. J. Exp. Med..

[11] Polson A.G., Zheng B., Elkins K., Chang W., Du C., Dowd P., Yen L., Tan C., Hongo J.-A., Koeppen H. (2006). Expression Pattern of the Human FcRH/IRTA Receptors in Normal Tissue and in B-Chronic Lymphocytic Leukemia. Int. Immunol..

[12] Rostamzadeh D., Kazemi T., Amirghofran Z., Shabani M. (2018). Update on Fc Receptor-like (FCRL) Family: New Immunoregulatory Players in Health and Diseases. Expert Opin. Ther. Targets.

[13] Mamidi M.K., Huang J., Honjo K., Li R., Tabengwa E.M., Neeli I., Randall N.L., Ponnuchetty M.V., Radic M., Leu C.-M. (2023). FCRL1 Immunoregulation in B Cell Development and Malignancy. Front. Immunol..

[14] Harwood N.E., Batista F.D. (2010). Early Events in B Cell Activation. Annu. Rev. Immunol..

[15] Xu Y., Harder K.W., Huntington N.D., Hibbs M.L., Tarlinton D.M. (2005). Lyn Tyrosine Kinase: Accentuating the Positive and the Negative. Immunity.

[16] Monroe J.G. (2006). ITAM-Mediated Tonic Signalling through Pre-BCR and BCR Complexes. Nat. Rev. Immunol..

[17] Johnson S.A., Pleiman C.M., Pao L., Schneringer J., Hippen K., Cambier J.C. (1995). Phosphorylated Immunoreceptor Signaling Motifs (ITAMs) Exhibit Unique Abilities to Bind and Activate Lyn and Syk Tyrosine Kinases. J. Immunol. Baltim. Md. 1950.

[18] Yasuda T., Tezuka T., Maeda A., Inazu T., Yamanashi Y., Gu H., Kurosaki T., Yamamoto T. (2002). Cbl-b Positively Regulates Btk-Mediated Activation of Phospholipase C-Gamma2 in B Cells. J. Exp. Med..

[19] Kurosaki T., Tsukada S. (2000). BLNK: Connecting Syk and Btk to Calcium Signals. Immunity.

[20] Ishiai M., Kurosaki M., Pappu R., Okawa K., Ronko I., Fu C., Shibata M., Iwamatsu A., Chan A.C., Kurosaki T. (1999). BLNK Required for Coupling Syk to PLC Gamma 2 and Rac1-JNK in B Cells. Immunity.

[21] Kurosaki T. (2002). Regulation of B-Cell Signal Transduction by Adaptor Proteins. Nat. Rev. Immunol..

[22] Srinivasan L., Sasaki Y., Calado D.P., Zhang B., Paik J.H., DePinho R.A., Kutok J.L., Kearney J.F., Otipoby K.L., Rajewsky K. (2009). PI3 Kinase Signals BCR-Dependent Mature B Cell Survival. Cell.

[23] Wang J., Xu L., Shaheen S., Liu S., Zheng W., Sun X., Li Z., Liu W. (2017). Growth of B Cell Receptor Microclusters Is Regulated by PIP2 and PIP3 Equilibrium and Dock2 Recruitment and Activation. Cell Rep..

[24] Tolar P. (2017). Cytoskeletal Control of B Cell Responses to Antigens. Nat. Rev. Immunol..

[25] Fleire S.J., Goldman J.P., Carrasco Y.R., Weber M., Bray D., Batista F.D. (2006). B Cell Ligand Discrimination through a Spreading and Contraction Response. Science.

[26] Xu L., Auzins A., Sun X., Xu Y., Harnischfeger F., Lu Y., Li Z., Chen Y.-H., Zheng W., Liu W. (2015). The Synaptic Recruitment of Lipid Rafts Is Dependent on CD19-PI3K Module and Cytoskeleton Remodeling Molecules. J. Leukoc. Biol..

[27] DeLuca J.M., Murphy M.K., Wang X., Wilson T.J. (2021). FCRL1 Regulates B Cell Receptor-Induced ERK Activation through GRB2. J. Immunol. Baltim. Md. 1950.

[28] Yousefi Z., Sharifzadeh S., Yar-Ahmadi V., Andalib A., Eskandari N. (2019). Fc Receptor-Like 1 as a Promising Target for Immunotherapeutic Interventions of B-Cell-Related Disorders. Biomark. Insights.

[29] Chen X., Li G., Wan Z., Liu C., Zeng Y., Liu W. (2015). How B Cells Remember? A Sophisticated Cytoplasmic Tail of mIgG Is Pivotal for the Enhanced Transmembrane Signaling of IgG-Switched Memory B Cells. Prog. Biophys. Mol. Biol..

[30] Chen X., Pan W., Sui Y., Li H., Shi X., Guo X., Qi H., Xu C., Liu W. (2015). Acidic Phospholipids Govern the Enhanced Activation of IgG-B Cell Receptor. Nat. Commun..

[31] Maeda F.Y., van Haaren J.J., Langley D.B., Christ D., Andrews N.W., Song W. (2021). Surface-Associated Antigen Induces Permeabilization of Primary Mouse B-Cells and Lysosome Exocytosis Facilitating Antigen Uptake and Presentation to T-Cells. eLife.

[32] Natkanski E., Lee W.-Y., Mistry B., Casal A., Molloy J.E., Tolar P. (2013). B Cells Use Mechanical Energy to Discriminate Antigen Affinities. Science.

[33] Spillane K.M., Tolar P. (2018). Mechanics of Antigen Extraction in the B Cell Synapse. Mol. Immunol..

[34] Du X., Nagata S., Ise T., Stetler-Stevenson M., Pastan I. (2008). FCRL1 on Chronic Lymphocytic Leukemia, Hairy Cell Leukemia, and B-Cell Non-Hodgkin Lymphoma as a Target of Immunotoxins. Blood.

[35] Yousefi Z., Sharifzadeh S., Zare F., Eskandari N. (2023). Fc Receptor-like 1 (FCRL1) Is a Novel Biomarker for Prognosis and a Possible Therapeutic Target in Diffuse Large B-Cell Lymphoma. Mol. Biol. Rep..

[36] Kazemi T., Asgarian-Omran H., Hojjat-Farsangi M., Shabani M., Memarian A., Sharifian R.A., Razavi S.M., Jeddi-Tehrani M., Rabbani H., Shokri F. (2008). Fc Receptor-like 1-5 Molecules Are Similarly Expressed in Progressive and Indolent Clinical Subtypes of B-Cell Chronic Lymphocytic Leukemia. Int. J. Cancer.

[37] Nicolson N.L., Talpaz M., Nicolson G.L. (1996). Chromatin Nucleoprotein Complexes Containing Tightly Bound C-Abl, P53 and Bcl-2 Gene Sequences: Correlation with Progression of Chronic Myelogenous Leukemia. Gene.

[38] Jackson T.A., Haga C.L., Ehrhardt G.R.A., Davis R.S., Cooper M.D. (2010). FcR-like 2 Inhibition of B Cell Receptor-Mediated Activation of B Cells. J. Immunol..

[39] Li F.J., Schreeder D.M., Li R., Wu J., Davis R.S. (2013). FCRL3 Promotes TLR9-Induced B-Cell Activation and Suppresses Plasma Cell Differentiation. Eur. J. Immunol..

[40] Jourdan M., Robert N., Cren M., Thibaut C., Duperray C., Kassambara A., Cogné M., Tarte K., Klein B., Moreaux J. (2017). Characterization of Human FCRL4-Positive B Cells. PLoS ONE.

[41] Kim C.C., Baccarella A.M., Bayat A., Pepper M., Fontana M.F. (2019). FCRL5+ Memory B Cells Exhibit Robust Recall Responses. Cell Rep..

[42] Tolnay M. (2022). Lymphocytes Sense Antibodies through Human FCRL Proteins: Emerging Roles in Mucosal Immunity. J. Leukoc. Biol..

[43] Davis R.S., Wang Y.-H., Kubagawa H., Cooper M.D. (2001). Identification of a Family of Fc Receptor Homologs with Preferential B Cell Expression. Proc. Natl. Acad. Sci. USA.

[44] Won W.-J., Foote J.B., Odom M.R., Pan J., Kearney J.F., Davis R.S. (2006). Fc Receptor Homolog 3 Is a Novel Immunoregulatory Marker of Marginal Zone and B1 B Cells. J. Immunol..

[45] Ehrhardt G.R.A., Hsu J.T., Gartland L., Leu C.-M., Zhang S., Davis R.S., Cooper M.D. (2005). Expression of the Immunoregulatory Molecule FcRH4 Defines a Distinctive Tissue-Based Population of Memory B Cells. J. Exp. Med..

[46] Falini B., Tiacci E., Pucciarini A., Bigerna B., Kurth J., Hatzivassiliou G., Droetto S., Galletti B.V., Gambacorta M., Orazi A. (2003). Expression of the IRTA1 Receptor Identifies Intraepithelial and Subepithelial Marginal Zone B Cells of the Mucosa-Associated Lymphoid Tissue (MALT). Blood.

[47] Masuda K., Davis R.S., Maruyama T., Zhang J., He T., Cooper M.D., O-Wang J., Burrows P.D. (2005). FcRY, an Fc Receptor Related Gene Differentially Expressed during B Lymphocyte Development and Activation. Gene.

[48] Masir N., Jones M., Pozzobon M., Marafioti T., Volkova O.Y., Mechetina L.V., Hansmann M.-L., Natkunam Y., Taranin A.V., Mason D.Y. (2004). Expression Pattern of FCRL (FREB, FcRX) in Normal and Neoplastic Human B Cells. Br. J. Haematol..

[49] Shabani M., Bayat A.A., Jeddi-Tehrani M., Rabbani H., Hojjat-Farsangi M., Ulivieri C., Amirghofran Z., Baldari C.T., Shokri F. (2014). Ligation of Human Fc Receptor Like-2 by Monoclonal Antibodies down-Regulates B-Cell Receptor-Mediated Signalling. Immunology.

[50] Li F.J., Ding S., Pan J., Shakhmatov M.A., Kashentseva E., Wu J., Li Y., Soong S., Chiorazzi N., Davis R.S. (2008). FCRL2 Expression Predicts IGHV Mutation Status and Clinical Progression in Chronic Lymphocytic Leukemia. Blood.

[51] Liang X., Du L., Fan Y. (2023). The Potential of FCRL Genes as Targets for Cancer Treatment: Insights from Bioinformatics and Immunology. Aging.

[52] Nückel H., Collins C.H., Frey U.H., Sellmann L., Dürig J., Siffert W., Dührsen U. (2009). FCRL2 mRNA Expression Is Inversely As-sociated with Clinical Progression in Chronic Lymphocytic Leukemia. Eur. J. Haematol..

[53] Xu M., Zhao R., Cao H., Zhao Z.J. (2002). SPAP2, an Ig Family Receptor Containing Both ITIMs and ITAMs. Biochem. Biophys. Res. Commun..

[54] Cui X., Liu C.-M., Liu Q.-B. (2020). FCRL3 Promotes IL-10 Expression in B Cells through the SHP-1 and P38 MAPK Signaling Pathways. Cell Biol. Int..

[55] Sohn H.W., Krueger P.D., Davis R.S., Pierce S.K. (2011). FcRL4 Acts as an Adaptive to Innate Molecular Switch Dampening BCR Signaling and Enhancing TLR Signaling. Blood.

[56] Ehrhardt G.R.A., Hijikata A., Kitamura H., Ohara O., Wang J.-Y., Cooper M.D. (2008). Discriminating Gene Expression Profiles of Memory B Cell Subpopulations. J. Exp. Med..

[57] Ikeda J., Kohara M., Tsuruta Y., Nojima S., Tahara S., Ohshima K., Kurashige M., Wada N., Morii E. (2017). Immunohistochemical Analysis of the Novel Marginal Zone B-Cell Marker IRTA1 in Malignant Lymphoma. Hum. Pathol..

[58] Falini B., Agostinelli C., Bigerna B., Pucciarini A., Pacini R., Tabarrini A., Falcinelli F., Piccioli M., Paulli M., Gambacorta M. (2012). IRTA1 Is Selectively Expressed in Nodal and Extranodal Marginal Zone Lymphomas. Histopathology.

[59] Zhu Z., Davis R. (2010). Fc Receptor-like 5 Has Dominant Inhibitory Function in B Cells That Is Mediated via Lyn and SHP-1 (84.5). J. Immunol..

[60] Davis R.S. (2015). B-1 Cell Development and Function. Ann. N. Y. Acad. Sci..

[61] Haga C.L., Ehrhardt G.R.A., Boohaker R.J., Davis R.S., Cooper M.D. (2007). Fc Receptor-like 5 Inhibits B Cell Activation via SHP-1 Tyrosine Phosphatase Recruitment. Proc. Natl. Acad. Sci. USA.

[62] Ise T., Maeda H., Santora K., Xiang L., Kreitman R.J., Pastan I., Nagata S. (2005). Immunoglobulin Superfamily Receptor Translocation Associated 2 Protein on Lymphoma Cell Lines and Hairy Cell Leukemia Cells Detected by Novel Monoclonal Antibodies. Clin. Cancer Res..

[63] Ise T., Nagata S., Kreitman R.J., Wilson W.H., Wayne A.S., Stetler-Stevenson M., Bishop M.R., Scheinberg D.A., Rassenti L., Kipps T.J. (2007). Elevation of Soluble CD307 (IRTA2/FcRH5) Protein in the Blood and Expression on Malignant Cells of Patients with Multiple Myeloma, Chronic Lymphocytic Leukemia, and Mantle Cell Lymphoma. Leukemia.

[64] Dement-Brown J., Newton C.S., Ise T., Damdinsuren B., Nagata S., Tolnay M. (2012). Fc Receptor-like 5 Promotes B Cell Proliferation and Drives the Development of Cells Displaying Switched Isotypes. J. Leukoc. Biol..

[65] Franco A., Kraus Z., Li H., Seibert N., Dement-Brown J., Tolnay M. (2018). CD21 and FCRL5 Form a Receptor Complex with Robust B-Cell Activating Capacity. Int. Immunol..

[66] Rostamzadeh D., Dabbaghmanesh M.H., Shabani M., Hosseini A., Amirghofran Z. (2015). Expression Profile of Human Fc Receptor-like 1, 2, and 4 Molecules in Peripheral Blood Mononuclear Cells of Patients with Hashimoto’s Thyroiditis and Graves’ Disease. Horm. Metab. Res..

[67] Baranov K.O., Volkova O.I., Mechetina L.V., Chikaev N.A., Reshetnikova E.S., Nikulina G.M., Taranin A.V., Naiakshin A.M. (2012). Expression of Human B-Cell Specific Receptor FCRL1 in Normal Individuals and in Patients with Autoimmune Diseases. Mol. Biol..

[68] Khanzadeh A., Habibagahi Z., Hosseini A., Amirghofran Z. (2016). Investigation of the Human FCRL1, 2, and 4 Gene Expressions in Patients with Rheumatoid Arthritis. Rheumatol. Int..

[69] Zhao S.-X., Liu W., Zhan M., Song Z.-Y., Yang S.-Y., Xue L.-Q., Pan C.-M., Gu Z.-H., Liu B.-L., Wang H.-N. (2013). A Refined Study of FCRL Genes from a Genome-Wide Association Study for Graves’ Disease. PLoS ONE.

[70] Wang K., Pei H., Huang B., Yang R.-L., Wu H.-Y., Zhu X., Zhu L. (2012). Overexpression of Fc Receptor-like 1 Associated with B-Cell Activation during Hepatitis B Virus Infection. Braz. J. Med. Biol. Res..

[71] Terrier B., Nagata S., Ise T., Rosenzwajg M., Pastan I., Klatzmann D., Saadoun D., Cacoub P. (2014). CD21−/Low Marginal Zone B Cells Highly Express Fc Receptor–like 5 Protein and Are Killed by Anti–Fc Receptor–like 5 Immunotoxins in Hepatitis C Virus–Associated Mixed Cryoglobulinemia Vasculitis. Arthritis Rheumatol..

[72] Zhong Z., Shi D., Xiao M., Fu D., Feng S., Kong Q., Li J., Li Z. (2021). Expression Profile of Fc Receptor-like Molecules in Patients with IgA Nephropathy. Hum. Immunol..

[73] Saadoun D., Terrier B., Bannock J., Vazquez T., Massad C., Kang I., Joly F., Rosenzwajg M., Sene D., Benech P. (2013). Expansion of Autoreactive Unresponsive CD21-/Low B Cells in Sjögren’s Syndrome Associated Lymphoproliferation. Arthritis Rheumatol..

[74] Kochi Y., Yamada R., Suzuki A., Harley J.B., Shirasawa S., Sawada T., Bae S.-C., Tokuhiro S., Chang X., Sekine A. (2005). A Functional Variant in FcRH3, Encoding Fc Receptor Homolog 3, Is Associated with Rheumatoid Arthritis and Several Autoimmunities. Nat. Genet..

[75] Sullivan R.T., Kim C.C., Fontana M.F., Feeney M.E., Jagannathan P., Boyle M.J., Drakeley C.J., Ssewanyana I., Nankya F., Mayanja-Kizza H. (2015). FCRL5 Delineates Functionally Impaired Memory B Cells Associated with Plasmodium Falciparum Exposure. PLOS Pathog..

[76] Portugal S., Tipton C.M., Sohn H., Kone Y., Wang J., Li S., Skinner J., Virtaneva K., Sturdevant D.E., Porcella S.F. (2015). Malaria-Associated Atypical Memory B Cells Exhibit Markedly Reduced B Cell Receptor Signaling and Effector Function. Elife.

[77] Poonia B., Ayithan N., Nandi M., Masur H., Kottilil S. (2018). HBV Induces Inhibitory FcRL Receptor on B Cells and Dysregulates B Cell-T Follicular Helper Cell Axis. Sci. Rep..

[78] Doi H., Tanoue S., Kaplan D.E. (2014). Peripheral CD27-CD21- B-Cells Represent an Exhausted Lymphocyte Population in Hepatitis C Cirrhosis. Clin. Immunol..

[79] Portugal S., Obeng-Adjei N., Moir S., Crompton P.D., Pierce S.K. (2017). Atypical Memory B Cells in Human Chronic Infectious Diseases: An Interim Report. Cell. Immunol..

[80] Capone M., Bryant J.M., Sutkowski N., Haque A. (2016). Fc Receptor-like Proteins in Pathophysiology of B-Cell Disorder. J. Clin. Cell. Immunol..

[81] Jenks S.A., Cashman K.S., Zumaquero E., Marigorta U.M., Patel A.V., Wang X., Tomar D., Woodruff M.C., Simon Z., Bugrovsky R. (2018). Distinct Effector B Cells Induced by Unregulated Toll-like Receptor 7 Contribute to Pathogenic Responses in Systemic Lupus Erythematosus. Immunity.

[82] Kulemzin S.V., Zamoshnikova A.Y., Yurchenko M.Y., Vitak N.Y., Najakshin A.M., Fayngerts S.A., Chikaev N.A., Reshetnikova E.S., Kashirina N.M., Peclo M.M. (2011). FCRL6 Receptor: Expression and Associated Proteins. Immunol. Lett..

[83] Schreeder D.M., Pan J., Li F.J., Vivier E., Davis R.S. (2008). FCRL6 Distinguishes Mature Cytotoxic Lymphocytes and Is Upregulated in Patients with B Cell Chronic Lymphocytic Leukemia. Eur. J. Immunol..

[84] Davis R.S. (2020). Roles for the FCRL6 Immunoreceptor in Tumor Immunology. Front. Immunol..

[85] Wilson T.J., Presti R.M., Tassi I., Overton E.T., Cella M., Colonna M. (2007). FcRL6, a New ITIM-Bearing Receptor on Cytolytic Cells, Is Broadly Expressed by Lymphocytes Following HIV-1 Infection. Blood.

[86] Mavilio D., Lombardo G., Kinter A., Fogli M., La Sala A., Ortolano S., Farschi A., Follmann D., Gregg R., Kovacs C. (2006). Characterization of the Defective Interaction between a Subset of Natural Killer Cells and Dendritic Cells in HIV-1 Infection. J. Exp. Med..

[87] Zhang L., Ma S., Wang H., Su H., Su K., Li L. (2017). Identification of Pathogenic Genes Related to Rheumatoid Arthritis through Integrated Analysis of DNA Methylation and Gene Expression Profiling. Gene.

[88] Liu J., Jiang Y., Liu J., Tian C., Lin Y., Yang Y., Zhang Z., Fang Y., Huang B., Lin H. (2024). Fc Receptor-like a Promotes Malignant Behavior in Renal Cell Carcinoma and Correlates with Tumor Immune Infiltration. Cancer Med..

[89] Wang X., Lin R., Zeng Y., Wang Y., Wei S., Lin Z., Chen S., Ye Z., Chen L. (2022). High Expression of FCRLB Predicts Poor Prognosis in Patients with Colorectal Cancer. Front. Genet..

[90] Hosen N. (2024). Identification of Cancer-Specific Cell Surface Targets for CAR-T Cell Therapy. Inflamm. Regen..

[91] Cappell K.M., Kochenderfer J.N. (2023). Long-Term Outcomes Following CAR T Cell Therapy: What We Know so Far. Nat. Rev. Clin. Oncol..

[92] Chen J., Qiu S., Li W., Wang K., Zhang Y., Yang H., Liu B., Li G., Li L., Chen M. (2023). Tuning Charge Density of Chimeric Antigen Receptor Optimizes Tonic Signaling and CAR-T Cell Fitness. Cell Res..

[93] Mukherjee M., Mace E.M., Carisey A.F., Ahmed N., Orange J.S. (2017). Quantitative Imaging Approaches to Study the CAR Immunological Synapse. Mol. Ther. J. Am. Soc. Gene Ther..

[94] Ledbetter J.A., Rabinovitch P.S., June C.H., Song C.W., Clark E.A., Uckun F.M. (1988). Antigen-Independent Regulation of Cytoplasmic Calcium in B Cells with a 12-kDa B-Cell Growth Factor and Anti-CD19. Proc. Natl. Acad. Sci. USA.

[95] Krop I., Shaffer A.L., Fearon D.T., Schlissel M.S. (1996). The Signaling Activity of Murine CD19 Is Regulated during Cell Development. J. Immunol. Baltim. Md. 1950.

[96] Giampaolo S., Wójcik G., Klein-Hessling S., Serfling E., Patra A.K. (2019). B Cell Development Is Critically Dependent on NFATc1 Activity. Cell. Mol. Immunol..

[97] Doody G.M., Billadeau D.D., Clayton E., Hutchings A., Berland R., McAdam S., Leibson P.J., Turner M. (2000). Vav-2 Controls NFAT-Dependent Transcription in B- but Not T-Lymphocytes. EMBO J..

[98] Bucher P., Erdmann T., Grondona P., Xu W., Schmitt A., Schürch C., Zapukhlyak M., Schönfeld C., Serfling E., Kramer D. (2020). Targeting Chronic NFAT Activation with Calcineurin Inhibitors in Diffuse Large B-Cell Lymphoma. Blood.

[99] Parker K.R., Migliorini D., Perkey E., Yost K.E., Bhaduri A., Bagga P., Haris M., Wilson N.E., Liu F., Gabunia K. (2020). Single-Cell Analyses Identify Brain Mural Cells Expressing CD19 as Potential Off-Tumor Targets for CAR-T Immunotherapies. Cell.

[100] Sotillo E., Barrett D.M., Black K.L., Bagashev A., Oldridge D., Wu G., Sussman R., Lanauze C., Ruella M., Gazzara M.R. (2015). Convergence of Acquired Mutations and Alternative Splicing of CD19 Enables Resistance to CART-19 Immunotherapy. Cancer Discov..

[101] Orlando E.J., Han X., Tribouley C., Wood P.A., Leary R.J., Riester M., Levine J.E., Qayed M., Grupp S.A., Boyer M. (2018). Genetic Mechanisms of Target Antigen Loss in CAR19 Therapy of Acute Lymphoblastic Leukemia. Nat. Med..

[102] Asnani M., Hayer K.E., Naqvi A.S., Zheng S., Yang S.Y., Oldridge D., Ibrahim F., Maragkakis M., Gazzara M.R., Black K.L. (2020). Retention of CD19 Intron 2 Contributes to CART-19 Resistance in Leukemias with Subclonal Frameshift Mutations in CD19. Leukemia.

[103] Ledererova A., Dostalova L., Kozlova V., Peschelova H., Ladungova A., Culen M., Loja T., Verner J., Pospisilova S., Smida M. (2021). Hypermethylation of CD19 Promoter Enables Antigen-Negative Escape to CART-19 in Vivo and in Vitro. J. Immunother. Cancer.

[104] Fitzgerald K.N., Quesada A.E., von Keudell G., Raj S., Lewis N.E., Dogan A., Salles G., Palomba M.L. (2022). CD19 Epitope Masking by Tafasitamab Leads to Delays in Subsequent Use of CD19 CAR T-Cell Therapy in Two Patients with Aggressive Mature B-Cell Lymphomas. Leuk. Lymphoma.

[105] Liao W., Kohler M.E., Fry T., Ernst P. (2021). Does Lineage Plasticity Enable Escape from CAR-T Cell Therapy? Lessons from MLL-r Leukemia. Exp. Hematol..

[106] Jacoby E., Nguyen S.M., Fountaine T.J., Welp K., Gryder B., Qin H., Yang Y., Chien C.D., Seif A.E., Lei H. (2016). CD19 CAR Immune Pressure Induces B-Precursor Acute Lymphoblastic Leukaemia Lineage Switch Exposing Inherent Leukaemic Plasticity. Nat. Commun..

[107] Gardner R., Wu D., Cherian S., Fang M., Hanafi L.-A., Finney O., Smithers H., Jensen M.C., Riddell S.R., Maloney D.G. (2016). Acquisition of a CD19-Negative Myeloid Phenotype Allows Immune Escape of MLL-Rearranged B-ALL from CD19 CAR-T-Cell Therapy. Blood.

[108] Li L.-Z., Sun Q., Fang Y., Yang L.-J., Xu Z.-Y., Hu J.-H., Cao L., Huang J.-Y., Hong M., Li J.-Y. (2020). A Report on Lineage Switch at Relapse of CD19 CAR-T Therapy for Philadelphia Chromosome-Positive B-Precursor Acute Lymphoblastic Leukemia. Chin. Med. J..

[109] Sharma P., Kanapuru B., George B., Lin X., Xu Z., Bryan W.W., Pazdur R., Theoret M.R. (2022). FDA Approval Summary: Idecabtagene Vicleucel for Relapsed or Refractory Multiple Myeloma. Clin. Cancer Res. Off. J. Am. Assoc. Cancer Res..

[110] Cho S.-F., Lin L., Xing L., Li Y., Yu T., Anderson K.C., Tai Y.-T. (2020). BCMA-Targeting Therapy: Driving a New Era of Immunotherapy in Multiple Myeloma. Cancers.

[111] Sanchez E., Gillespie A., Tang G., Ferros M., Harutyunyan N.M., Vardanyan S., Gottlieb J., Li M., Wang C.S., Chen H. (2016). Soluble B-Cell Maturation Antigen Mediates Tumor-Induced Immune Deficiency in Multiple Myeloma. Clin. Cancer Res. Off. J. Am. Assoc. Cancer Res..

[112] Sanchez E., Li M., Kitto A., Li J., Wang C.S., Kirk D.T., Yellin O., Nichols C.M., Dreyer M.P., Ahles C.P. (2012). Serum B-Cell Maturation Antigen Is Elevated in Multiple Myeloma and Correlates with Disease Status and Survival. Br. J. Haematol..

[113] Carpenter R.O., Evbuomwan M.O., Pittaluga S., Rose J.J., Raffeld M., Yang S., Gress R.E., Hakim F.T., Kochenderfer J.N. (2013). B-Cell Maturation Antigen Is a Promising Target for Adoptive T-Cell Therapy of Multiple Myeloma. Clin. Cancer Res. Off. J. Am. Assoc. Cancer Res..

[114] Seipel K., Porret N., Wiedemann G., Jeker B., Bacher V.U., Pabst T. (2022). sBCMA Plasma Level Dynamics and Anti-BCMA CAR-T-Cell Treatment in Relapsed Multiple Myeloma. Curr. Issues Mol. Biol..

